# Minimally invasive, non-ablative Er:YAG laser treatment of stress urinary incontinence in women—a pilot study

**DOI:** 10.1007/s10103-016-1884-0

**Published:** 2016-02-09

**Authors:** Nikola Fistonić, Ivan Fistonić, Štefica Findri Guštek, Iva Sorta Bilajac Turina, Ingrid Marton, Zdenko Vižintin, Marko Kažič, Irena Hreljac, Tadej Perhavec, Matjaž Lukač

**Affiliations:** Ob/Gyn Clinic, University Hospital Merkur, Zagreb, Croatia; Ob/Gyn and Menopause Clinic, Zagreb, Croatia; Medical School University of Rijeka, Rijeka, Croatia; Department of Obstetrics and Gynecology, General Hospital Sveti Duh, Zagreb, Croatia; Fotona d.o.o., Stegne 7, 1000 Ljubljana, Slovenia

**Keywords:** Stress urinary incontinence, Er:YAG laser, Minimally invasive SUI therapy

## Abstract

The study presents an assessment of mechanism of action and a pilot clinical study of efficacy and safety of the Er:YAG laser for the treatment of stress urinary incontinence (SUI). The subject of this study is a treatment of SUI with a 2940 nm Er:YAG laser, operating in a special SMOOTH mode designed to increase temperature of the vaginal mucosa up to maximally 60–65 °C without ablating the epidermis. Numerical modelling of the temperature distribution within mucosa tissue following an irradiation with the SMOOTH mode Er:YAG laser was performed in order to determine the appropriate range of laser parameters. The laser treatment parameters were further confirmed by measuring in vivo temperatures of the vaginal mucosa using a thermal camera. To investigate the clinical efficacy and safety of the SMOOTH mode Er:YAG laser SUI treatment, a pilot clinical study was performed. The study recruited 31 female patients suffering from SUI. Follow-ups were scheduled at 1, 2, and 6 months post treatment. ICIQ-UI questionnaires were collected as a primary trial endpoint. Secondary endpoints included perineometry and residual urine volume measurements at baseline and all follow-ups. Thermal camera measurements have shown the optimal increase in temperature of the vaginal mucosa following treatment of SUI with a SMOOTH mode Er:YAG laser. Primary endpoint, the change in ICIQ-UI score, showed clinically relevant and statistically significant improvement after all follow-ups compared to baseline scores. There was also improvement in the secondary endpoints. Only mild and transient adverse events and no serious adverse events were reported. The results indicate that non-ablative Er:YAG laser therapy is a promising minimally invasive non-surgical option for treating women with SUI symptoms.

## Introduction

Female stress urinary incontinence [1] is a significant public health problem, with estimated prevalence rates ranging from 4–35 % of adult women [2].

The initial treatment option for SUI is pelvic floor muscle training (PFMT), which produces good results, but often fails to achieve consistent long-term improvement because of poor patient compliance and lack of support and guidance [3, 4]. Traditional surgical approaches such as open or laparoscopic colposuspension and anterior vaginal repair have been largely replaced with less-invasive midurethral sling suspension procedures, which have popularized the surgical option of treating SUI among patients [5, 6]. While midurethral sling procedures are highly effective, they are associated with adverse events and complications such as bleeding, bladder perforation, urethral injury, infection, and groin pain [7]. In recent years, single incision polypropylene mesh mini-slings have been introduced and have become very popular due to the minimal invasiveness of the procedure. However, they have since been associated with frequent side effects, prompting the FDA in 2012 to require extensive post-market studies for all marketed mini-slings [8]. This has resulted in increased caution among patients considering SUI surgery and in a search for new, safer procedures among physicians and the industry.

It is well known that using laser energy to achieve heat pulsing (i.e., temporarily increasing the temperature) of collagen can improve collagen structure and initiate neo-collagenesis [9, 10]. As a result of the temperature increase, intermolecular cross-links that stabilize collagen triple-helix structure are broken, which leads to the shrinkage of collagen fibrils and improvement in tissue firmness.

Collagen is an important component of pelvic floor supportive structures—it makes up more than 80 % of protein content of the endopelvic fascia. Childbirth trauma can lead to destruction of collagen fibers in the pelvic floor, while aging slows down the synthesis of new collagen, both resulting in decreasing collagen content [11]. It was shown that pubocervical fasciae of incontinent women have a low collagen content [12] and also that stress urinary incontinence is more frequent in women with reduced collagen content in their anterior vaginal walls [13].

The above facts support the idea that laser-mediated heat pulsing of the endopelvic fascia and pelvic floor tissue could represent an effective non-surgical method for treating female urinary incontinence and other disorders resulting from diminished pelvic floor support.

One existing treatment for SUI that most resembles the mechanism of action of laser therapy is the treatment of SUI using radiofrequency (RF) emitting devices. Although RF methods are less invasive than surgery, transurethral treatment comes with a need for anesthesia and increased risk of post-operative pain and urinary tract infections [14–17].

Here, we investigate a novel, minimally invasive alternative SUI therapy that is based on thermally inducing reinforcement of the pelvic floor using an infra-red Er:YAG laser. The Er:YAG laser wavelength of 2.94 mm is strongly absorbed in water which is the major constituent of human soft tissue. A precisely controlled sequence(s) of sub-ablative Er:YAG laser pulses are delivered to the intravaginal mucous tissue in order to achieve controlled heating of the collagen in the deeper mucosa layers, without over-heating of the mucosa surface.

The aim of the present study was to assess the efficacy of the new laser treatment in achieving the optimal rise in the temperature of the anterior vaginal wall that supports the urethra and to assess the clinical outcome of this new non-invasive laser treatment for mild-to-severe stages of female stress urinary incontinence.

## Materials and methods

### Laser system

Fotona Dynamis laser manufactured by Fotona d.d., Slovenia, incorporating an Er:YAG laser with enabled SMOOTH mode was used in this study. The “SMOOTH mode pulse” with a duration of 250 msec consisted of a fast sequence of six individual SP mode (300 μsec) micro pulses with intra-pulse temporal separation of 50 msec. Full beam handpiece R11 and patterned (pixelated) handpiece PS03 together with the gynecological adapters (G-set) were used to deliver Er:YAG laser energy onto the vaginal mucous tissue. The Fotona handpiece PS03 is equipped with a pixel screen, resulting in the overall laser spot having a patterned (dotted) internal beam structure, with the centers of individual circular beam dots of diameter of 0.85 mm being separated by approximately 2 mm. The motivation behind the design of the patterned handpiece PS03 is to make the treatment less invasive by reducing the treatment area to isolated beam islands.

### Numerical model

In this study, we use a previously developed numerical model [18], to determine the influence of laser parameters on the desired treatment end effects in the treated tissue. The numerical model consists of three main stages: laser energy absorption, heat diffusion deeper into the tissue, and heat convection into the surrounding air. The thermodynamic behavior of tissue water, which is the major absorber of Er:YAG (2.94 μm) laser irradiation, is combined with the elastic response of the surrounding solid medium. The tissue is treated as a two component medium, consisting of microscopic spherical cavities where water is trapped in a non-absorbing elastic medium. Heat absorption is modeled by taking into account nonlinear water absorption coefficient, which is decreased by deposited energy laser irradiation [19].

The boundaries of the tissue are on the top side of cylindrical computational domain subjected to convection [20]. Other edges of computational domain are thermally insulated using Neumann boundary condition, which prevents the heat to be lost through the edges.

The diffusion of heat is introduced into the model using three-dimensional unsteady heat conduction equation in cylindrical coordinates with heat-source term

$$ \overset{.}{q}=\left(1-{\mu}_r\right){\mu}_aI $$

from the absorbed laser irradiation [21]:

$$ \frac{1}{r}\frac{\partial }{\partial r}\left(r\frac{\partial T}{\partial r}\right)+\frac{\partial^2T}{\partial {z}^2}+\frac{\overset{.}{q}}{\lambda }=\frac{\rho c}{\lambda}\frac{\partial T}{\partial t} $$

where *μ*_*r*_ is the coefficient of reflection at tissue surface, *μ*_*a*_ the absorption coefficient, *I * the depth distribution of intensity of incident laser beam, *T* the tissue temperature, *λ* the thermal conductivity of the tissue, *ρ* the density, and *c* the specific heat modified by the nonlinear model of Majaron et al. [18]. The same parameters were used in this study.

### Thermal camera measurements to assess heating of the mucosal tissue

Thermal camera (ThermaCAM P45, Flir, USA) measurements were made of the mucosa surface temperatures following irradiation of the introitus area with the Fotona Dynamis Er:YAG Laser System. Measurements were made following irradiation of the introitus with the R11 (full-beam, used in the first two steps of the Incontilase® procedure (see below “Pilot clinical study” for protocol details) and PS03 (patterned handpiece, used in the third step of the Incontilase® procedure) handpieces. Both handpieces were set to a 7 mm overall spot size, and the SMOOTH mode pulse was selected with a fluence of 3, 6, and 10 J/cm^2^ (resulting in micro pulse fluences of 0.5, 1, and 1.67 J/cm^2^, respectively). Up to four consecutive SMOOTH pulses were applied to the vaginal mucosa at the repetition rate of 1.6 Hz.

No alteration to the commercial laser device was made. The laser was fired as in a normal operation by pressing the footswitch, and the camera image was recorded following an emitted laser pulse. Measurement accuracy as according to the camera manufacturer was ±2 °C.

Since the camera software assumes a uniform body temperature, the measured temperatures represent a weighted average of the mucosa temperature within the penetration depth of the detected thermal radiation of approximately 50 μm.

### Pilot clinical study

This pilot study included 31 patients suffering from stress urinary incontinence.

The inclusion criteria for recruitment to the study were history of vaginal delivery, stress urinary incontinence, normal cell cytology, negative urine culture, no injuries and bleeding in the vaginal canal, introitus and vestibule.

The exclusion criteria were severe prolapse and damage of the recto-vaginal fascia, patients with urge incontinence, patients with severe neurological conditions associated with incontinence (multiple sclerosis, spinal cord injury, stroke, Parkinson’s disease), neurogenic bladder, insulin-dependent diabetes mellitus, actual urinary tract infection, hematuria, age ≤18 and >70 years, pregnancy, less than 24 weeks after vaginal delivery, body mass index (BMI) >30, intake of photosensitive drugs, injury or/and active infection in the treatment area, and undiagnosed vaginal bleeding.

The study was approved by the Ethics committee of the University of Rijeka School of Medicine in Rijeka, Croatia, and was carried out in Ob/Gyn Menopause Clinic in Zagreb, Croatia.

The procedure was performed with a 2940 nm Er:YAG laser (XS Dynamis, Fotona, Slovenia), using a special modality, SMOOTH mode, which delivers laser energy in a non-ablative, thermal-only manner, based on the pulsing sequence designed to achieve deep heating of the vaginal mucosa to around 60 °C. Protocol was done according to manufacturer’s instructions (Incontilase® application notes, Fotona). The patients were placed in lithotomy position, and laser probes, consisting of a specially designed speculum and a laser delivery handpiece were introduced into the vaginal canal. Briefly, the protocol consisted of three consecutive steps, during which the laser irradiation was applied to anterior vaginal wall (PS03 patterned beam, four consecutive SMOOTH pulses with a fluence of 10 J/cm^2^), the whole circumference of vaginal canal (R11 full beam, four consecutive SMOOTH mode pulses with a fluence of 3 J/cm^2^), and vestibule area (PS03 patterned beam, four consecutive SMOOTH mode pulses with a fluence of 3 J/cm^2^), respectively. Several passes of laser energy were applied to each area. On average, cumulative energy applied in all three steps reached from 2500–3000 J, depending on the length of the vaginal canal. The average treatment duration was approximately 10 min. Patient discomfort, treatment tolerability, and potential adverse events were monitored during laser treatment. No anesthesia was used before or during the session. Patients were instructed to avoid increased intra-abdominal pressure and refrain from sexual intercourse for 14 days after intervention. First follow-up appointments were scheduled at 1 month, the second follow-up at 2 months, and the third follow-up at 6 months after the intervention.

The degree of incontinence and its impact on quality of life (QoL) were assessed with the International Consultation on Incontinence Questionnaire-Urinary Incontinence Short Form (ICIQ-UI SF) [22], where a maximum score of 21 represents permanent incontinence. The questionnaire allows for the assessment of the prevalence, frequency, and perceived cause of urinary incontinence and its impact on everyday life. The results of the ICIQ-UI SF may be divided into the following four severity categories: mild (1–5), moderate (6–12), severe (13–18), and very severe (19–21) [22]. The residual urine volume was measured at each follow-up immediately after the patient returned from the washroom. Measurements were performed with a DC-8 ultrasound unit (Mindray, China). For the measurement of muscle strength of the pelvic diaphragm, an Apimedis perineometer (EXTT-101, Korea) was used to determine maximal and average pressure (mmHg) and mean duration of contractions (seconds).

Level of statistical significance was set to *p* < 0.05 and all confidence intervals were given at 95 % level. In all instances, two-tailed tests of statistical significance were used. The absolute and relative differences between medians at baseline and at second follow-up measurement were given. Statistical significance of the difference between mean outcomes values at baseline and at follow-up measurements was done by one-way ANOVA with Dunn’s multiple comparison tests. To compensate for high loss to follow-up, a last observation carried forward (LOCF) sensitivity analysis was done The assumption of the LOCF is that the patients lost to follow-up remained at the same level measured at their last appointment (either baseline or previous follow-up).

All statistical analyses were performed using GraphPad Prism statistical software.

## Results

### Numerical modelling of thermal penetration depth

**Fig 1:**
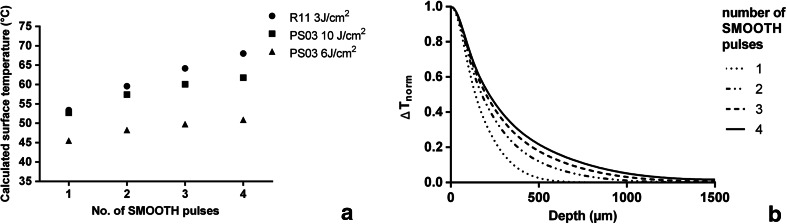
**a** Calculated temperature on the tissue surface following laser treatment with the PS03 handpiece for two different SMOOTH mode fluences (6 and 10 J/cm^2^) and with the R11 handpiece (fluence = 3 J/cm^2^), as a function of the number of consecutively delivered SMOOTH mode pulses. **b** Calculated normalized temperature change distribution in relation to depth below tissue surface following laser irradiation as a function of the number of consecutively delivered SMOOTH mode Er:YAG pulses using the handpiece PS03 at a SMOOTH mode pulse fluence of 10 J/cm^2^

Numerical modelling was carried out for laser parameters and conditions as used during thermal camera measurements. The calculations confirmed that no tissue ablation occurs at the Incontilase® laser treatment parameters. In order to compare predictions of the theoretical model with thermal camera measurements, Fig. 1a shows calculated average temperatures of a 50-μm thick superficial tissue layer, 20 msec following laser irradiation. It is clear that spatially local temperature accumulation after exposure to full-beam handpiece R11 is markedly higher than after exposure to the patterned handpiece PS03.

Figure 1b shows calculated normalized tissue temperature distributions in the longitudinal direction, for the patterned handpiece, and a SMOOTH mode pulse fluence of 10 J/cm^2^, as a function of the number of SMOOTH mode pulses delivered sequentially at a repetition rate of 1.6 Hz.

As can be seen, “heat pumping”, i.e., sequential irradiation of tissue with appropriately temporally separated laser pulses results in a temperature distribution several hundred microns deep within the tissue, without overheating of the surface temperature.

### Thermal camera measurements to assess heating of the mucosal tissue and thermal penetration depth calculation

**Fig. 2:**
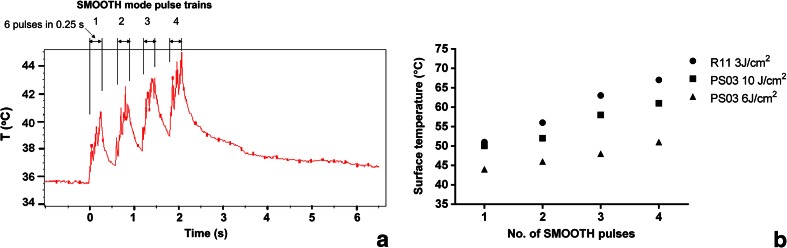
**a** An example of the measured temperature development on the introitus mucous tissue during four consecutively delivered SMOOTH mode Er:YAG (Fotona Dynamis XS) laser pulses (PS03 handpiece, SMOOTH mode pulse fluence: 3 J/cm^2^, total fluence delivered: 4 × 3 J/cm^2^ = 12 J/cm^2^). **b** Measured temperature on the introitus mucosa following laser treatment with the PS03 handpiece for two different SMOOTH mode fluences (6 and 10 J/cm^2^) and with the R11 handpiece (fluence = 3 J/cm^2^), as a function of the number of consecutively delivered SMOOTH mode pulses

Figure 2a shows the measured temporal evolution of the tissue temperature during an irradiation with a sequence of four SMOOTH mode pulses. From the graph, the temporary heat accumulation can be observed as an overall increase of temperature after each SMOOTH mode pulse.

Figure 2b shows the measured tissue surface temperature, as a function of the number of consecutively delivered SMOOTH mode pulses, as obtained with the PS03 handpiece, for SMOOTH mode pulse fluences of 3, 6, and 10 J/cm^2^, and with the R11 handpiece for a fluence of 3 J/cm^2^. In agreement with the theoretical model, for the same delivered laser fluence, the patterned beam handpiece results in a lower local temperature increase than the full beam handpiece.

### Pilot clinical study

Total of 31 women were included into the study. Their mean age was 46.6 years with the mean body mass index of 23.2 (Table 1). All patients (100 %) have completed the first follow-up. At 2nd follow-up, 2 months after the intervention, 17 (56.7 %) patients remained in the study and 14 (43.3 %) were lost to follow-up. At the third follow up, 6 months after intervention, 14 (43.3 %) patients remained in the study, while 17 (56.7 %) were lost to follow-up. Baseline age, parity, average birth weight, and body mass index of those who have remained in the study at later follow-ups were similar to those at the baseline. It appears that later lost to follow-up has not been associated with baseline values of any of the outcomes (Table 1).

**Table 1:** All patients’ characteristics at the baseline and baseline characteristics of those who remained into the study at the 1st and at the 2nd and 3rd follow-up

	Baseline *n* = 31	1st follow-up *n* = 31	2nd follow-up *n* = 17	3rd follow-up *n* = 14
Age	46.8 ± 9.1	46.8 ± 9.1	46.4 ± 9	46.7 ± 7.2
Parity	2 (1.7–3)	2 (1.7–3)	2 (2–3)	2 (1–3)
Average birth weight (kg)	3.5 ± 0.5	3.5 ± 0.5	3.5 ± 0.6	3.5 ± 0.5
BMI	23.3 ± 2.7	23.3 ± 2.7	23.8 ± 3.2	24.14 ± 3
BMI, *n* (%)				
Normal (≤24.9)	23 (74.2)	23 (74.2)	10 (58.8)	8 (57.1)
Overweight (25.0–29.9)	8 (25.8)	8 (25.8)	7 (41.2)	6 (42.9)
Obese (≥30.0)	0	0	0	0
Total	31 (100)	31 (100)	17 (100)	14 (100)
ICIQ-UI	12.9 ± 5	12.9 ± 5	12.5 ± 4.7	14 ± 3.7
Residual urine	3.8 (1.1–10.5)	3.8 (1.1–10.5)	3 (1.9–9.9)	5.6 (1.4–13)
Perineometry				
Maximal (mmHg)	9 (7.5–11.9)	9 (7.5–11.9)	9.7 (7.6–12.2)	8.4 (7.6–12.1)
Average (mmHg)	4.6 (3.8–6.4)	4.6 (3.8–6.4)	5.2 (3.9–6.7)	4.6 (4–6.2)
Duration (seconds)	10.7 (5.6–23.9)	10.7 (5.6–23.9)	10 (6.1–21.7)	10.4 (6.1–21.7)

Data are presented as medians (intra-quartile range), means ± SD, depending on the normality of the data,  or *n* (%), where patient distributions were calculated

*BMI* body mass index (body weight in kg divided by body height in m^2^)

No serious adverse events were observed or reported throughout the course of laser treatment and the follow-up period. All patients experienced sensation of warmth or teasing during treatment. Most patients reported increased vaginal discharge in the next few days after the procedure and slight vulvar edema, which disappeared within 48 h after the treatment. The Visual Analogue Scale (VAS, 0–10) pain level of 2 was reported  by 5 % of participants, while 95 % had score 0.

**Fig. 3:**
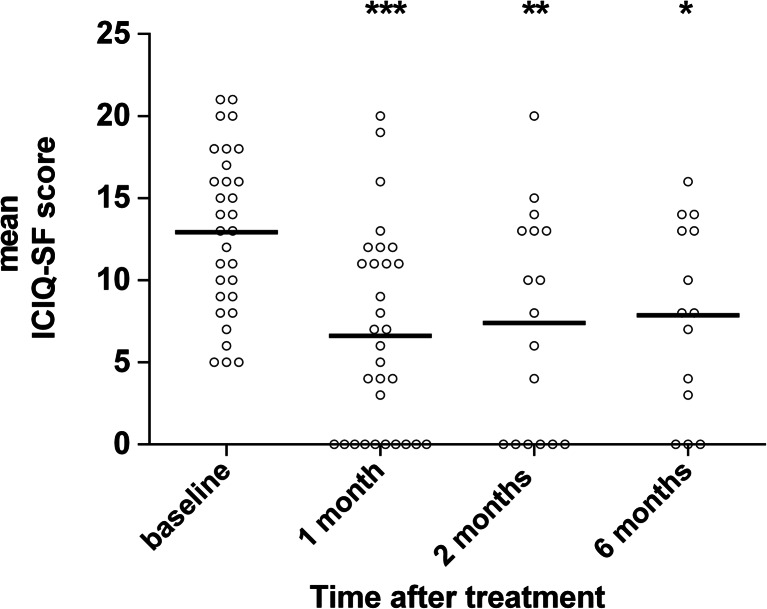
Mean scores from the ICIQ-UI questionnaire, which was the primary effectiveness endpoint, at baseline, and at 1, 2, and 6 months after treatment. *Circles* represent all patients that have participated in the measurement at each follow-up. * comparison test—***p < 0.001; **p < 0.01; *p < 0.05

The ICIQ-UI score, which was the primary efficacy endpoint of the study, was significantly decreased in all follow-ups compared to baseline. The score was decreased on average by 6.3 points after 1 month, by 5.3 points after 2 months, and by 5.1 points 6 months after treatment (Fig. 3 and Table 2). At baseline, all participants had urinary incontinence; 3/31 (9.7 %) had mild, 11/31 (35.5 %) moderate, 13/31 (41.9 %) severe, and 4/31 (12.9 %) suffered from very severe urinary incontinence. At the first follow-up, 1 month after the intervention, number of those with ICIQ-UI score = 0 indicating no urinary incontinence increased to 10/31 (32.52 %) (Table 2).

**Table 2:** Differences from baseline before the intervention, at 1st, 2nd, and 6th month follow-up

	Baseline (*n* = 31)	1-month follow-up (*n* = 31)			2-month follow-up (*n* = 17)			6-month follow-up (*n* = 14)		
			*P* value	AD		*P* value	AD		*P* value	AD
ICIQ-UI	12.9 ± 5	6.6 ± 6.1***	<0.001	−6.3	7.6 ± 6.7**	0.005	−5.3	7.8 ± 5.7*	0.01	−5.1
Residual urine	3.8 (1.1–10.5)	0.6 (0.1–3.3)*	0.01	−3.2	0.4 (0.2–3.7)*	0.04	−3.4	1.0 (0.2–3.3)	0.07	−2.8
Perineometry										
Maximal (mmHg)	9 (7.5–11.9)	11 (8.5–16.4)	0.36	2	12.2 (9.9–24.8)	0.06	3.2	11.5 (9.7–21)	0.18	2.5
Average (mmHg)	4.6 (3.8–6.4)	6 (4.7–9.5)	0.12	1.4	6 (4.5–15.2)	0.16	1.4	5.7 (4.5–14.7)	0.18	1.1
Duration (seconds)	10.7 (5.6–23.9)	15.4 (9.3–28.9)	0.75	4.7	29 (11.2–32.7)	0.15	18.3	27 (10.5–46)	0.09	16.3

Data are presented as mean ± SD or medians (interquartile ranges), depending on the normality of the data

*P P* value (one-way ANOVA, multiple comparison; or Kruskal-Wallis test, depending on the normality of the datasets), *AD* absolute difference in medians at baseline and follow-up, * indicates statistical significance

***p < 0.001; **p < 0.01; *p < 0.05

The LOCF sensitivity analysis has revealed statistically significant and clinically relevant differences after the intervention (Table 3).

**Table 3:** Sensitivity analysis. Differences from baseline before the intervention, at 1st, 2nd, and 6th month follow-up under the assumption that the 2nd and 6th months follow-up values for all those that were lost for follow-up were the same as at last measured follow-up

	Baseline (*n* = 31)	1-month follow-up (*n* = 31)			2-month follow-up (*n* = 31)			6-month follow-up (*n* = 31)		
			*P* value	AD		*P* value	AD		*P* value	AD
ICIQ-UI	12.9 ± 5	6.6 ± 6.1***	<0.001	−6.3	6.9 ± 6.3***	<0.001	−5.3	7 ± 6***	<0.001	−5.9
Residual urine	3.8 (1.1–10.5)	0.6 (0.1–3.3)*	0.02	−3.2	0.8 (0.25–3.6)*	0.03	−3.4	1.0 (0.2–3.5)**	0.007	−2.8
Perineometry										
Maximal (mmHg)	9 (7.5–11.9)	11 (8.5–16.4)	0.19	2	11.5 (10.1–20.2)*	0.02	2.5	12 (9.7–40)*	0.02	3
Average (mmHg)	4.6 (3.8–6.4)	6 (4.7–9.5)	0.06	1.4	6.1 (4.6–11.9)*	0.02	1.5	5.8 **(**4.5–12.4)*	0.03	1.2
Duration (seconds)	10.7 (5.6–23.9)	15.4 (9.3–28.9)	0.4	4.7	18.5 (9.5–29.8)	0.2	7.8	16.2 (10.5–36.2)	0.09	5.5

Data are presented as mean ± SD or medians (interquartile ranges), depending on the normality of the data

*P* one-way ANOVA, Dunn’s multiple comparison test, statistical significance between baseline and follow-up, *AD* absolute difference in medians between baseline and follow up, * indicates statistical significance

***p < 0.001; **p < 0.01; *p < 0.05

There was a statistically significant decrease in post void residual urine volume at all the first two follow-ups compared to baseline measurement (Table 2).

Perineometry measurements have shown a trend of improvement, but not statistically significant compared to baseline (Table 2).

However, the LOCF analysis of both post void residual volume and perineometry measurements showed significant improvement after treatment (Table 3).

## Discussion

Our theoretical calculations and in vivo thermal camera measurements have demonstrated that treatment with the SMOOTH mode Er:YAG laser at Incontilase® laser parameters results in a non-ablative temperature increase which is within the temperature window required to achieve collagen contraction and to initiate neocollagenesis in the vaginal mucosa in vivo*.* Published data shows that the shortening of the collagen fibrils without irreversible denaturation of their structure requires that temperatures do not exceed the optimal temperature range of 60 to 70 °C [23]. Both, numerical modelling and in vivo thermal camera imaging show that the Incontilase® treatment parameters result in the temperature increase of vaginal wall mucosa up to approximately 65 °C. Therefore, the Incontilase® treatment increases the mucosa temperature up to the optimal temperature range, but does not exceed the temperature threshold for surface ablation or irreversible denaturation of collagen.

The Er:YAG laser radiation acts almost like a surface heater due to the extremely shallow optical penetration depth of its mid-infrared wavelength and relies on heat diffusion to affect deeper layers of the mucosa. Since at sub-ablative fluences (F < 2 J/cm^2^) of a single Er:YAG pulse the thermally affected layer is below 10 μm, the laser procedure described in this study (Incontilase®) utilizes repetitive stacking of sub-ablative Er:YAG laser pulses, which can result in a tenfold increase in the depth of thermally affected soft tissue [24]. The concept of non-ablative pulse stacking of Er:YAG laser pulses has been already successfully applied in other medical areas, such as dermatology [25, 26].

We have used numerical modelling to calculate the spatial and temporal temperature distribution within the tissue during and after exposure to four SMOOTH mode pulses (as used in the Incontilase® method).

Repetitive SMOOTH pulsing resulted in heat deposition and temperature rise that reached several hundred microns below tissue surface (Figs. 1 and 2). These results are in agreement with those by Majaron et al. [18], whose numerical calculations predict deep tissue heating and resulting coagulation depths of above 100 μm for the irradiation of soft tissue with a train of Er:YAG pulses with parameters comparable to those of the procedure used in this study (SMOOTH mode).

Our calculations are also supported by histologic investigations using an in vivo rat skin model from Majaron et al. [27], which have confirmed prior theoretical predictions by demonstrating that a train of 3–10 Er:YAG pulses, delivered at 10–33 Hz repetition rate, and with the total full beam delivered fluence of up to 10 J/cm^2^, could achieve non-ablative coagulation of collagen deeper than 200 μm below the epidermal-dermal junction.

Further, histological data from a clinical study of Er:YAG eyelid treatment by Drnovsek-Olup et al. [26] that used similar laser parameters (Fotona SMOOTH mode, full beam, cumulative fluence 2 J/cm^2^) as are used in the present study, has shown deep (200 μm) collagen denaturation and skin regeneration without ablation of epidermis.

The rationale for using the patterned handpiece PS03 comes from fractional photothermolysis, the relatively new method of skin rejuvenation [28, 29]. Unlike standard full beam treatments, where the whole of the selected target area is irradiated, fractional photothermolysis seeks to only affect certain zones within the selected target area, producing tiny dot- or pixel-like treated areas in the skin. This leaves the other zones within the skin perfectly intact; hence, only causing fractional effect through the heat of the light source. The skin heals much faster than if the whole area was treated, as the ‘healthy’ untreated tissue surrounding the treated zones helps to fill in the affected area with new cells.

As the numerical model and thermal camera measurements have demonstrated, at the same fluence, treatment with the full beam handpiece R11 results in a higher local vaginal tissue temperature increase in comparison to treatment with the patterned handpiece PS03. This is because when the patterned PS03 handpiece is used, the heat diffuses both longitudinally into the deeper tissue layers, and transversally into the un-irradiated volume between the pixels. Because of this higher thermal diffusion in the transverse direction, the local temperature achieved after irradiation with PS03 handpiece at 10 J/cm^2^ is nearly the same as the one after irradiation with the full beam R11 handpiece at 3 J/cm^2.^

In order to show that the new approach in treating SUI is effective and safe, a pilot clinical trial of Incontilase® SUI treatment was performed.

The treatment resulted in clinically meaningful and statistically significant improvement in the SUI symptoms according to the primary efficacy endpoint—the ICIQ-UI questionnaire. The improvement was first observed at the first follow-up, 1 month after the treatment, and has remained stable to the third follow-up, 6 months after treatment. ICIQ-SF score at all follow-ups was improved by more than five points. Recently, a minimum important difference in the ICIQ-SF, indicating a clinically meaningful improvement, was determined to be 2.5 points [30]. The improvement of ICIQ-UI score was strongest at the first follow-up and slightly smaller at each subsequent follow-up. Although the clinically meaningful effect was endured, it might be beneficial to repeat the treatment at least once in order to fortify the effect.

There was significant decrease in the residual urine volume after treatment; the effect remained strong at all follow-ups, indicating improved voiding efficiency following treatment.

Perineometry measurements included maximum pressure, average pressure, and pressure duration of pelvic floor muscle contraction. All parameters showed a trend of increase, which was not statistically significant. These results were somewhat expected, as the increases in perineometry measurements indicate some increased support to pelvic floor muscles, which is not dramatic, since the laser therapy does not directly act on pelvic floor muscles. It would be interesting to combine laser therapy with PFMT and evaluate the effect of the combined non-invasive treatments, as simultaneous muscle exercise combined with laser reinforcement of the connective tissue could bring synergistic benefits to pelvic floor reinforcement.

The treatment was proven to be safe—all of the observed side effects (most frequent were edema and stronger vaginal discharge) were mild and of transient nature. The Er:YAG laser SUI treatment as described in the present study is minimally invasive—the laser thermal energy in the form of SMOOTH pulse sequences is delivered intra-vaginally, with the emphasis on the anterior vaginal wall, which supports the bladder and urethra that lie above. There is no need for anesthesia, since only moderate increase of temperature is achieved without any surface ablation. Because the SMOOTH pulse sequences are pre-programmed to achieve the abovementioned moderate temperature rise, there is minimal risk of over-heating and thermal damage to the tissue.

Since laser treatment of stress urinary incontinence is a novel method, there were only a few pilot studies published on the use of laser for this indication, all showing promising results [31, 32]. Gaspar et al. [33] used a fractional CO2 laser for strengthening and tightening the vaginal mucosa and have measured a significant improvement in the same perineometry measurements that were done in the present study. While the indication was not the same, the mechanism of action of laser treatment for SUI is largely due to thickening and strengthening of the vaginal wall, with an emphasis to the anterior wall, which supports the bladder and urethra. Besides laser treatment, patients from the Gaspar et al. study also received intravaginally applied platelet-rich plasma and pelvic floor muscle exercise with biofeedback control, which explains the stronger perineometry effects and gives further arguments for future trials of combined effects of laser and PMFT.

We recognize that the main limitations of the pilot study are the lack of a control group, high loss to follow-up, and the relatively short follow-up time. However, the study was designed as an exploratory pilot study and the presented results are promising as well as extremely useful in design of future, larger randomized control trials. High loss to follow-up is most likely due to the lack of incentive and geographical dispersion of the patients. It is interesting to note that at the first follow-up, which was completed by all patients, there was a marked improvement in the severity scale, showing that almost a third of patients scored 0, meaning no incontinence. Of these, 50 % were lost to both subsequent follow-ups, three completed only the 2-month follow-up, where the score remained 0, but then missed the 6-month follow-up. Two out of 10 patients that have scored 0 at the 1-month follow-up completed both 2 and 6-month follow-ups—both remained completely dry, with the score of 0. This indicates that patients with completely improved results might have lost incentive to further participate in the study, since there were no predicted incentives or mechanisms to keep patients enrolled throughout.

The differences in baseline values between responders and those that were lost to follow-up were quite small. In order to check the impact of the missing data on the results, sensitivity analysis was done, where last measured observation was carried forward to the subsequent missed follow-ups. All of the methods yielded comparable, clinically meaningful and statistically significant results of the primary effectiveness endpoint. The results were even better in the sensitivity analysis, once more confirming that the lost-to-follow-up patients were largely the ones with very much improved results at the first follow-up.

We conclude that the non-ablative SMOOTH mode Er:YAG intravaginal laser treatment induces deep thermal effect on the vaginal wall, which based on our pilot study seems to result in a clinically meaningful improvement of female SUI. The SMOOTH mode Er:YAG laser therapy is a promising minimally invasive, safe, and effective option for treating women with SUI symptoms. Randomized control trials are needed for further evaluation of this new SUI treatment.
